# Thoracic endometriosis syndrome at the University of Ilorin Teaching Hospital

**DOI:** 10.7196/AJTCCM.2018.v24i2.213

**Published:** 2018-06-21

**Authors:** Anthony Linegar

**Affiliations:** Head of Thoracic Surgery, University of Cape Town, Groote Schuur Hospital Affiliated Lecturer, University of the Free State, Universitas Hospital 206 Medical Chambers, N1 City Hospital, Goodwood, Cape Town

## Editorial

This issue of the *AJTCCM* includes a retrospective review by Adeoye *et al*. ^[1]^ on their experience with thoracic endometriosis syndrome, as seen over a 3.5-year period at a teaching hospital in Ilorin, Nigeria.

Thoracic endometriosis syndrome, which refers to the presence of endometrial tissue in the lung parenchyma or on the pleural surfaces, is an extremely rare condition. Presentation is variable, but patients can be broadly grouped into those who present with signs and symptoms related to catamenial pneumothorax, catamenial haemothorax or intrapulmonary (parenchymal or airway) nodules, which usually present with haemoptysis. In the reported study, pleural effusion was the most frequent presenting sign.

As with all rare conditions, diagnosis is often delayed. A diagnosis is based on a high clinical index of suspicion in the first instance. Pointers include: a cyclical presentation of chest pain, dyspnoea, cough or haemoptysis that occurs in relation to the menstrual cycle, cases presenting in women during the productive years and symptoms that affect the right hemithorax. Clinical suspicion should prompt investigation that involves a computed tomography scan and testing CA-125 serum levels.

The authors recognise the importance of thoracoscopic techniques for obtaining a histological diagnosis and intrathoracic management of the pleural space. They also mention the inadequacy of basing the diagnosis on pleural fluid and bronchial lavage cytology. Their observation that chemical pleurodesis alone has a poor success rate in patients with pleural effusions is also supported by the literature. They conclude that early thoracoscopic intervention is desirable, and that pleurectomy should replace pleurodesis when indicated.

The authors have provided a concise summary and literature review of various aspects of this interesting and complex condition.
